# Normal Bone Turnover in Transient Hyperphosphatasemia

**DOI:** 10.4274/Jcrpe.680

**Published:** 2012-09-11

**Authors:** Stepan Kutilek, Barbora Cervickova, Pavla Bebova, Marie Kmonickova, Vladimir Nemec

**Affiliations:** 1 University of Pardubice, Pardubice Hospital and Faculty of Health Studies, Pediatrics, Pardubice, Czech Republic

**Keywords:** Alkaline phosphatase, transient hyperphosphatasemia, bone turnover

## Abstract

Transient hyperphosphatasemia of infancy and early childhood (THI) is characterized by a temporary isolated elevation of serum alkaline phosphatase activity (ALP), predominantly its bone or liver isoform, in either sick or healthy children under 5 years of age. Return to normal ALP levels usually occurs within four months. Spontaneous rise of ALP might concern the physician, especially when treating seriously ill children. However, THI is considered a benign biochemical disorder with no clinical consequences. Some existing reports support the hypothesis that THI is a result of increased bone turnover. We present evidence of normal bone turnover in two children with THI. In a one-year-old girl and a boy of the same age, high ALP levels (31 and 109 μkat/L, respectively) were accidentally detected. The children had no signs of metabolic bone disease or of liver disease. The high ALP levels returned to normal in two months, thus fulfilling the diagnosis of THI. In both patients, serum parathyroid hormone and bone turnover markers, serum CrossLaps, and serum osteocalcin were neither elevated, nor did these markers follow the ALP dynamics, thus reflecting normal bone turnover in THI. Children with THI should be spared from extensive investigations and unnecessary vitamin D treatment.

**Conflict of interest:**None declared.

## INTRODUCTION

Transient hyperphosphatasemia of infancy and early childhood (THI) is characterized by transiently increased serum activity of alkaline phosphatase (ALP), predominantly of its bone or liver isoform, in children under 5 years of age. No signs or symptoms of metabolic bone disease or hepatic pathology related to increased ALP have been reported, nor is there any disease entity common for all children with THI. Furthermore, THI may occur in both healthy and sick children (1,2,3,4,5,6,7). Following the criteria devised by Kraut et al (1), the diagnosis of THI is based on: (i) age below 5 years; (ii) presence of various unrelated symptoms; (iii) no evidence of bone or liver disease; (iv) ALP analysis showing elevations in bone and/or liver activity; (v) return to normal ALP values in four months. Spontaneous rise of ALP may be of concern to the physician, especially when following a seriously ill child. However, THI is considered a benign biochemical disorder with no clinical consequences (1,2,3,4,5,6,7,8,9). Some reports support the hypothesis that THI is a result of increased bone turnover (9,10,11,12). We present evidence about normal bone turnover in two children with THI.

## CASE PRESENTATIONS

**Patient 1**

A 12-month-old female infant was referred for diarrhea and failure to thrive. She was a full-term baby with a birth weight of 2740 g (-1.6 SD) and birth length of 49 cm (-1.1 SD). At admission, her body weight was 6770 g (-2.6 SD), body length 68 cm (-2 SD), and body mass index (BMI) was 14.6 (-1.4 SD). Laboratory findings revealed high ALP activity (31.3 μkat/L; colorimetric assay; normal age-related range 2.5 - 9.5 μkat/L), with otherwise normal values for serum creatinine, aspartate aminotransferase (AST) and alanin aminotransferase (ALT), ruling out hepatic pathology. There were no rachitic changes on the wrist X-ray and serum calcium level (Ca) was normal (2.3 mmol/L; reference value 2.2-2.6 mmol/L). The same was true for serum phosphate levels (P; 1.8 mmol/L; normal 1.0 - 2.0 mmol/L), ruling out rickets. Blood count was normal. Cystic fibrosis and coeliac disease were ruled out by normal sweat chloride concentration (17.7 mmol/L) and non-significant tissue transglutaminase IgA antibody level (7 U/mL; normal 0-20 U/mL) with normal serum IgA levels. Microbiological evaluation of the stool samples, including rotavirus and adenovirus, was negative. Abdominal ultrasound was normal. The patient´s state improved and she was dismissed three days later with a body weight of 7140 g (-2.2 SD) and was reevaluated in a follow-up visit 10 days later. At that time, her ALP was still elevated (27 μkat/L; bone isoform 80%, liver isoform 20%), with normal values for AST, ALT, Ca (2.59 mmol/L), serum P (1.8 mmol/L), and serum parathyroid hormone (PTH; 34.3 pg/L; (Quest Diagnostics/ Nichols); normal 10-65 pg/L). The serum level of CrossLaps (CTx; marker of bone resorption) was normal (1311 ng/L; electrochemiluminescence immunoassay-ECLIA on Elecsys-Cobas analyzers; age-related normal value 202-2311 ng/L). The serum concentration of osteocalcin (OC; marker of bone formation) was also normal (154 ng/mL; ECLIA on Elecsys-Cobas analyzers; age-related normal range 40-160 ng/mL; adult range 11-43 ng/mL). Following another period of six weeks, the patient was doing well - her weight was 7850 g (-2.2 SD), body length 70 cm (-2.1 SD), and BMI was 16 (-0.67 SD). The ALP concentration was fully normalized (5.43 μkat/L). Ca (2.61 mmol/L) and P (1.76 mmol/L) levels were normal, while CTx and OC remained almost unchanged (1300 ng/L and 150 ng/mL, respectively), thus unrelated to the ALP dynamics.

**Patient 2**

This patient was a severely retarded boy with confirmed congenital rubella syndrome (brain cyst, microcephaly, microphthalmia, psychomotor retardation) who had a periodic laboratory check-up at the age of nine months, with normal values for ALP (4.17 μkat/L), Ca (2.4 mmol/L), and P (1.83 mmol/L). He had no history of trauma or fractures, nor was he taking any medication known to affect bone metabolism. At the next laboratory follow-up visit at age 12 months, the patient’s ALP peaked to 109 μkat/L. Ca (2.45 mmol/L), P (1.88 mmol/L) and bilirubin, AST and ALT levels were all normal at this time. Two weeks later, his ALP was 89.7 μkat/L (90% represented by bone isoform, 10% liver isoform), with otherwise normal values for indices of bone metabolism (Ca 2.25 mmol/L, P 1.78 mmol/L, OC 88.6 ng/mL, CTx 1150 ng/L, PTH 30 pg/L). AST and ALT were also within normal reference ranges. Six weeks later, his ALP dropped to 5.42 μkat/L, while other markers continued to remain within normal values (Ca 2.43 mmol/L, P 2.2 mmol/L, OC 102 ng/mL, CTx 1150 ng/L, PTH 46.6 pg/L). In the course of the hyperphosphatasemic episode, the patient showed no signs of acute gastrointestinal or respiratory infection, nor any signs of failure to thrive.

## DISCUSSION

ALP (EC 3.1.3.1) is a phosphohydrolase. It is a membrane-bound metallo-enzyme, responsible for removing P groups from many types of molecules, including nucleotides, proteins, and alkaloids. The ALP structure is determined both genetically and by post-translational modification. ALP is produced in the liver, bone, and the placenta, and is normally present in high concentrations in growing bone and in bile. There are three tissue-specific isoenzymes (intestinal, placental, and placental-like) and a tissue non-specific ubiquituous isoenzyme comprising hepatic, bone and renal isoforms. ALP is a routine marker in the diagnosis of hepatic disorders and metabolic bone diseases. In healthy adults, the major activity of ALP is represented by liver and bone isoforms, while in healthy infants and children, as a result of growth, the serum is rich in the bone isoform of ALP (3). The differential diagnosis of high ALP in childhood includes bone disorders (rickets, osteomalacia, healing fractures, juvenile Paget’s disease, bone tumors), hepatopathy (cholestasis, malignancy), kidney disease (chronic renal failure, renal tubular acidosis and other tubulopathies), drug ingestion (co-trimoxazol, antiepileptics) or THI. The peak value of ALP in THI is usually very high (5-30 fold above upper reference ranges) in comparison with other causes of ALP elevation (2,3,10). Both our patients fulfilled the criteria for THI devised by Kraut et al (1). While in the first patient, the THI was preceded and accompanied by diarrhoea and failure to thrive, the second patient was free of such symptoms. THI is a benign disorder believed to be triggered by an infectious disease, most probably viral, as described in siblings and in patients hospitalized together (2,3). In some patients, THI is preceded by a viral infection with predominantly gastrointestinal symptomatology and occasionally by transient failure to thrive. 

Increased production of ALP in its tissues of origin (most probably in bone and/or liver), increased activation of circulating ALP, and impaired clearance of ALP from the circulation have all been proposed as the mechanisms responsible for THI (2,3). Out of these hypotheses, the last one, namely impaired clearance of ALP from the circulation, is considered as the most probable. However, increased production of ALP received also some attention since isolated findings of increased serum activity of tartrate-resistant acid phosphatase (TRACP) and urinary hydroxyproline excretion in patients with THI supported the hypothesis of transiently increased bone turnover (9,10,11,12). Higher TRACP was detected in two patients with THI (9,10). Furthermore, mildly increased urinary hydroxyproline excretion in THI was observed in 1 of 5 children and in 11 out of 33 patients in two studies from Germany (12,13) and in 3 out of 5 infants in a Czech study (11). The increase in bone resorption is known to be closely coupled to bone formation and followed by increased osteoblast activity. It has been hypothesized that putative transient increase in bone resorption might have been triggered by a viral infection. Furthermore, the duration of THI (four months) resembles the duration of bone resorption cycle. It has been therefore postulated that in THI, the increased ALP activity, reflecting high bone formation, is preceded by an increased bone resorption, as evidenced by the increased urinary hydroxyproline excretion and/or high TRACP (9,10,11). However, the increase in bone resorption markers in THI was always only very mild, never reaching the magnitude of ALP. Furthermore, normal bone turnover, reflected by normal values of carboxyterminal pyridinoline-cross-linked telopeptide of type I collagen (ICTP) and border-line levels of free cross-links and carboxyterminal propeptide of type I collagen (PICP) was observed in the above-mentioned German study on 33 children with THI (12). None of the studies where increased urinary hydroxyproline was observed appear to have controlled for dietary collagen intake, which can influence urinary hydroxyproline levels (14). In addition, normal values of PTH have been reported in the following groups of children with THI: healthy ones (14,15), those with acute but mild illness (diarrhoea, respiratory tract infections) (15), and in patients after liver or kidney transplantation (16,17,18,19,20). Similarly, in our patients, the levels of OC, CTx, Ca, P and PTH were within normal ranges and not related to ALP dynamics, thus ruling out increased bone turnover. 

In conclusion, the hypothesis of increased bone turnover as being the sole cause of extremely high ALP in THI seems quite improbable. THI is a benign disorder, a biochemical rather than clinical abnormality, and has a good prognosis. Children with THI should be spared from extensive investigations and unnecessary vitamin D treatment attempts. 
